# Cor triatriatum and lipomatous hypertrophy of the interatrial septum in the elderly: a case report

**DOI:** 10.1186/1476-7120-8-4

**Published:** 2010-03-09

**Authors:** Pier Paolo Bassareo, Roberto Tumbarello, Giuseppe Mercuro

**Affiliations:** 1 Department of Cardiovascular and Neurological Sciences, University of Cagliari, Cagliari, Italy; 2 Division of Paediatric Cardiology, G. Brotzu Hospital, Cagliari, Italy

## Abstract

Cor triatriatum is a rare congenital heart defect characterized by the presence of a fibromuscular membrane dividing the left atrium into two distinct chambers. Lipomatous hypertrophy of the atrial septum is an infrequently observed benign abnormality caused by large fatty tissue deposits in the interatrial septum. An increased incidence of atrial arrhythmias is described in both pathologies, while a significant obstruction of blood flow mimicking mitral stenosis is typically manifested in cor triatriatum. We report the case of a 75-year-old woman with a previously undescribed association of the above stated abnormalities detected by both transthoracic and transeosophageal echocardiography. Diagnosis was confirmed by means of computed tomography. The singular physiologic and anatomic factors underlying survival until such a late age are described. The diagnostic, therapeutic and surgical management is discussed and a short review of the literature performed.

## Background

Cor triatriatum (CT), first described by Church in 1868, is a rare congenital heart disease (presenting approx. 0.4% of all congenital cardiac anomalies). It is characterized by the presence of a fibromuscular diaphragm which divides the left atrium into two chambers: a posterosuperior chamber receiving blood flow from the pulmonary veins and an anteroinferior chamber communicating with the mitral valve and the left atrial appendage. The two chambers generally communicate through one or more openings in the intra-atrial membrane [1-4]. Different classifications of CT have been provided, on the basis of the number and size of orifices in the membrane and of the anatomic shape of the accessory left atrial chamber (diaphragmatic, hour-glass and tubular) [5,6]. No unified embryologic theory for the aetiology of CT has yet been established. Most of specialists of this area believe that this disorder represents a failure in incorporation of the common pulmonary vein into the left atrium. However, several variants of CT feature have marked inconsistencies with this theory [7]. As a general rule, the defect becomes symptomatic in early infancy, although on rare occasions it may remain asymptomatic until adulthood, diagnosis being often purely incidental [8,9]. Literature reports describe approximately fifty cases detected in adulthood, at an age ranging from 16 to 76 years. Up to now, a total of approximately two hundred and fifty cases of CT have been described [10]. In patients with heart failure or pulmonary oedema surgery should be undertaken as soon as possible [11]. Lipomatous hypertrophy (LH) of the interatrial septum is a rare cardiac tumour, morphologically and pathohistologically well described: it is a benign deposition of fatty tissue within the interatrial septum, histologically characterized by a predominance of adult fat cells with interspersed hypertrophic cardiac muscle fibres. Occasionally, multivacuolar fat cells similar to fetal fat cells may be observed [12]. The first in vivo diagnosis of this type of tumour dates back to 1982 [13]. Diagnosis in live subjects is purely incidental, the majority of findings deriving from autoptic studies.

LH of the interatrial septum was first described by Prior in 1964, as a consequence of the detection of a non-encapsulated hypertrophic alteration of the normal structures of the interatrial septum [14]. This tumour must be differentiated from other types of lesions including myxomas, true cardiac lipomas, liposarcomas, parietal thrombi, metastatic tumours and amyloidosis that appear as septal tumour mass [15]. Approximately 200 cases have been reported in literature up to now. Most of the cases reported occurred in obese patients, particularly elderly women [16,17]. Clinical symptoms of the tumour are generally not specific or absent, although the incidence of supraventricular arrhythmias seems to be significantly increased [18]. Transthoracic and transoesophageal echocardiography and computed tomography imaging allow a simple non-invasive diagnosis [13,19,20]. Echocardiographic findings of a bi-lobed septal mass sparing the foramen ovale and feauturing a highly echogenic structure are invariably closely related to LH. Endomyocardial biopsy may be performed for a further evaluation of the mass.

Surgical therapy is used to treat LH of the interatrial septum only in patients displaying an obstruction of the superior caval vein or an intractable rhythm disturbance [21]. In our patient no significant obstruction of the superior caval vein was detected, although chronic fibrillation was observed. Due to the benign character of the tumour, physicians and surgeons decided against a complete resection.

If complete excision of the tumour is scheduled, reconstruction of the interatrial septum using either autologous pericardium or Dacron must be undertaken [21]. On the other hand, no tendency towards a rapid increase has been observed for LH of the interatrial septum. To this regard therefore, complete excision of the tumour is not strictly necessary. Moreover, neither partial nor total resection of the interatrial septum will provide relief from rhythm disorders. Here we report the case of an old woman with a not previously described association of the above stated abnormalities, concurring in developing the signs and symptoms of heart failure. The diagnostic detection and the singular physiologic and anatomic factors allowing survival at such a late age are described as well.

## Case presentation

### Medical history, symptoms, and signs

A 75-year-old female patient was referred to our clinical Centre following the onset of increasing dyspnea, fatigue and chronic fibrillation. The patient had a medical history of arterial hypertension treated with beta blockers and calcium antagonists and rheumatic arthritis.

Physical examination revealed an obese subject (height 151 cm; weight 74 Kg; BMI 32.4 Kg/m^2^). Systemic blood pressure was 132/87 and respiratory rate was 21 breaths per min. Upper venous congestion was visible from the pulse of jugular veins. Heart sounds were arrhythmic and wadded with a grade 1-2/6 systolic murmur over the left sternal edge. Basal and medium lungs auscultation revealed crackling wheezes.

### Instrumental tests

Electrocardiogram revealed atrial fibrillation with heart rate ranging from 70 to 100 beats/min.

**Figure 1:**
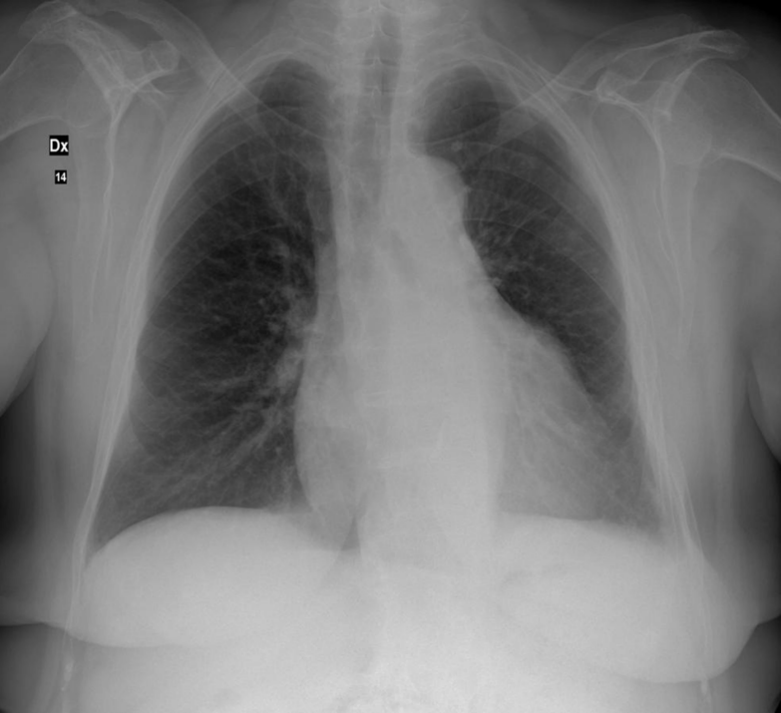
**Chest antero-posterior X-ray**.

Chest X-ray demonstrated an enlarged heart size, specially due to left atrium augmented profile, an aortic sclerosis, a previous left basal pleurisy and a more marked pulmonary vasculature (Figure 1).

**Figure 2:**
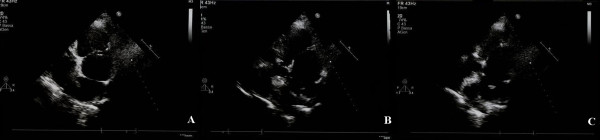
**Transthoracic echocardiogram: apical 4-chamber view showing a fibromuscular membrane subdividing the left atrium into proximal and distal chambers (PANEL A)**. Lipomatosis of the interatrial septum, with the typical "dumbbell" aspect is evident as well (PANEL B and C).

**Figure 3:**
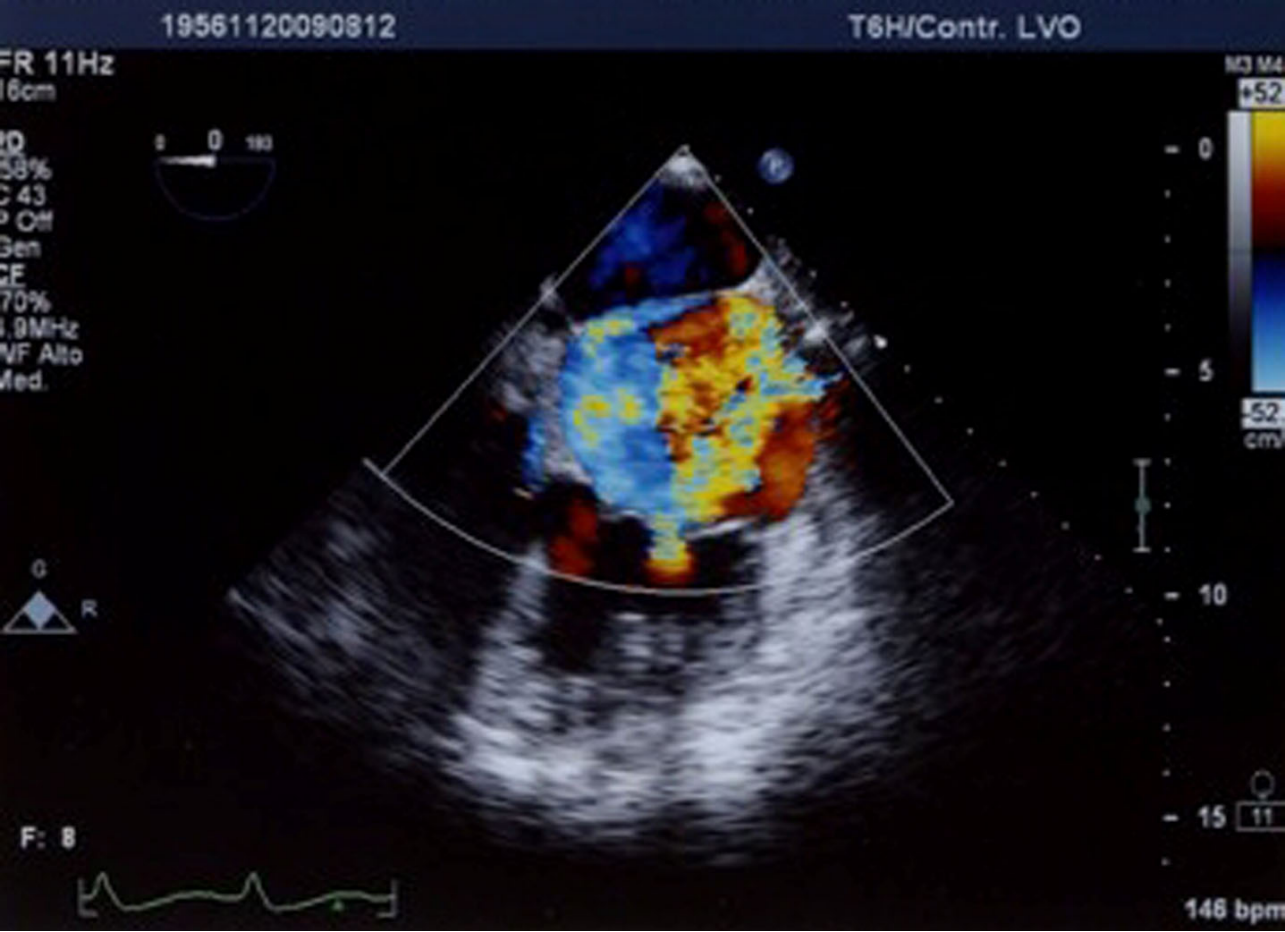
**Transoesophageal echocardiogram: turbulent colour flow across the diaphragm in the left atrium, suggestive of an insufficiency from a single central opening**.

**Figure 4:**
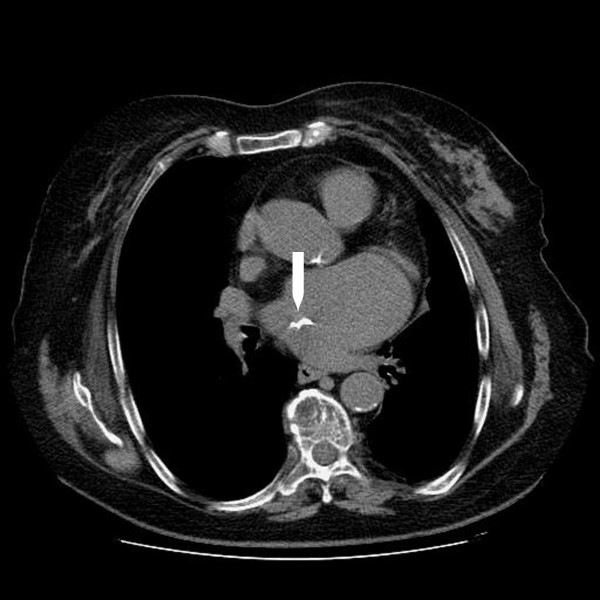
**Detection by computed tomography of lipomatous hypertrophy of the interatrial septum**.

**Figure 5:**
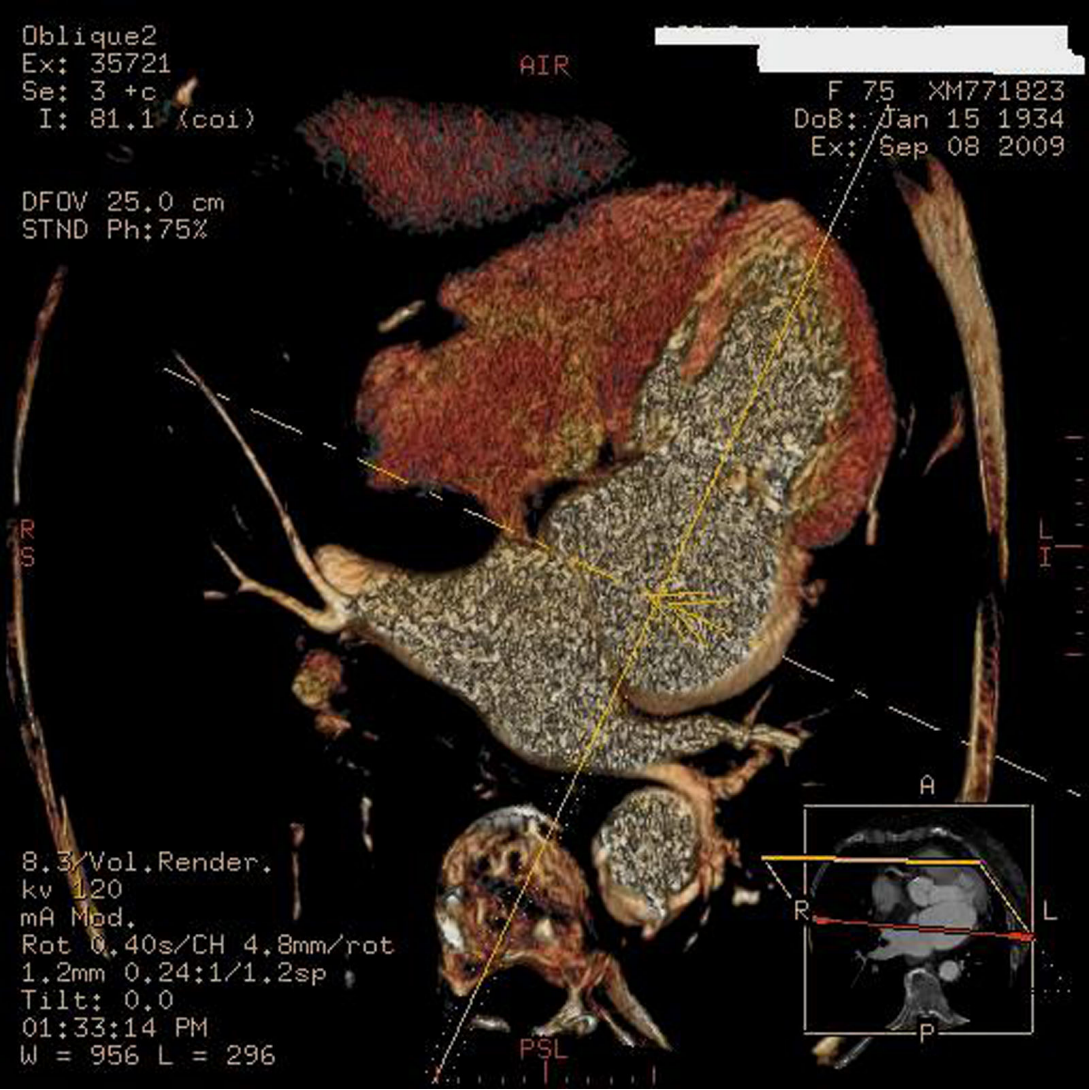
**Three dimensional reconstruction of computed tomography shows the fibromuscular membrane dividing the left atrium into two distinct chambers**.

Transthoracic echocardiography detected a membrane subdividing the dilated left atrium into two chambers and hypertrophy of the interatrial septum (Figure 2). Transeosophageal echocardiography also revealed the presence of the above stated diaphragm in the left atrium as well as echogenic hypertrophy of the interatrial septum (about 2 cm), sparing the foramen ovale. A turbulent flow across the membrane suggested that the orifice in the diaphragm was central. (Figure 3). Pulsed wave Doppler showed both severe stenosis (mean gradient 8 mmHg) and moderate insufficiency at the level of the orifice. No narrowing of the superior caval vein was observed. Diagnosis was confirmed by computed tomography of the chest and the anatomic substance at the level of interatrial septum was defined as fat (Figures 4 and 5). Magnetic resonance imaging was not performed being the patient claustrophobic. The latter technique, avoiding exposure to radiations, would represents an effective alternative to computed tomography. Coronary angiography demonstrated the presence of coronary artery disease, with 70% stenosis of the main stem, 75% stenosis of the left anterior descending artery and significant stenosis of the first diagonal branch.

### Surgical treatment

In the operative approach a median sternotomy was performed and an ascending aortic cannulation with bicaval venous return was applied. As a consequence of the identification of the pulmonary veins, an excision was made into the intra-atrial membrane (radial incision from the orifice of the diaphragm to the atrial septum). In addition, the most parts of fatty tissue mass were removed, and sent for urgent histological evaluation. Pathohistological examination of the resected tissue showed a pattern typical of LH Due to invasion of the tumour into the skeleton of the heart and histological findings of non-malignancy, no attempt was made to remove the tumour mass. Ultimately, subsequent to cardioplegic arrest of the heart, coronary artery bypass grafting was performed.

The postoperative course was uncomplicated and the patient was discharged from hospital one week after surgery with a saturation of 98%.

## Conclusions

In conclusion, this previously unreported association of CT and LH of the atrial septum contributed to the development of atrial fibrillation and subsequent heart failure. Clinical presentation of CT was delayed by the original probably large opening in the diaphragm. Subsequent fibrosis and calcification of the orifice manifested on ageing, and the development of both mitral stenosis and atrial fibrillation may account for the late conversion to a symptomatic state.

Transthoracic and transoesophageal echocardiography and computed tomography (or magnetic resonance imaging) represent the diagnostic tools of choice. Computed tomography was performed in order to better define the three-dimensional anatomy of the lesions, with a view to cardiac surgery. On the contrary, both transthoracic and transoesophageal echocardiography provide only bi-dimensional images. In this respect, three-dimensional echocardiography could be a potential alternative to computed tomography, being even more safe (no exposure to radiations) and cost-effective [22]. The technological advances made in this field may explain the recent increase in the reported frequency of the two pathologies described [23,24]. In case of hemodynamic instability or severe rhythm disorders a surgical correction must be taken into account.

## Abbreviations

CT: cor triatriatum; LH: lipomatous hypertrophy.

## Consent

Written informed consent was obtained from the patient for the publication of her case report and any accompanying images. A copy of the written consent is available for review by the Editor-in-Chief of this journal.

## Competing interests

The authors declare that they have no competing interests.

## Authors' contributions

PPB: acquisition of data, conception and design - RT: revising the manuscript critically - GM: final approval of the version to be published. All authors have read and approved the final manuscript.
